# Fluoxetine attenuates neuroinflammation in early brain injury after subarachnoid hemorrhage: a possible role for the regulation of TLR4/MyD88/NF-κB signaling pathway

**DOI:** 10.1186/s12974-018-1388-x

**Published:** 2018-12-20

**Authors:** Fu-yi Liu, Jing Cai, Chun Wang, Wu Ruan, Guo-ping Guan, Hai-zhou Pan, Jian-ru Li, Cong Qian, Jing-sen Chen, Lin Wang, Gao Chen

**Affiliations:** Department of Neurosurgery, The Second Affiliated Hospital of Zhejiang University School of Medicine, Hangzhou, China

**Keywords:** Subarachnoid hemorrhage, Early brain injury, Neuroinflammation, Fluoxetine

## Abstract

**Background:**

Neuroinflammation is closely associated with functional outcome in subarachnoid hemorrhage (SAH) patients. Our recent study demonstrated that fluoxetine inhibited NLRP3 inflammasome activation and attenuated necrotic cell death in early brain injury after SAH, while the effects and potential mechanisms of fluoxetine on neuroinflammation after SAH have not been well-studied yet.

**Methods:**

One hundred and fifty-three male SD rats were subjected to the endovascular perforation model of SAH. Fluoxetine (10 mg/kg) was administered intravenously at 6 h after SAH induction. TAK-242 (1.5 mg/kg), an exogenous TLR4 antagonist, was injected intraperitoneally 1 h after SAH. SAH grade, neurological scores, brain water content, Evans blue extravasation, immunofluorescence/TUNEL staining, quantitative real-time polymerase chain reaction (qRT-PCR), and western blot were performed.

**Results:**

Fluoxetine administration attenuated BBB disruption, brain edema, and improved neurological function after SAH. In addition, fluoxetine alleviated the number of Iba-1-positive microglia/macrophages, neutrophil infiltration, and cell death. Moreover, fluoxetine reduced the levels of pro-inflammatory cytokines, downregulated the expression of TLR4 and MyD88, and promoted the nuclear translocation of NF-κB p65, which *were* also found in rats with TAK-242 administration. Combined administration of fluoxetine and TAK-242 did not enhance the neuroprotective effects of fluoxetine.

**Conclusion:**

Fluoxetine attenuated neuroinflammation and improved neurological function in SAH rats. The potential mechanisms involved, at least in part, TLR4/MyD88/NF-κB signaling pathway.

**Electronic supplementary material:**

The online version of this article (10.1186/s12974-018-1388-x) contains supplementary material, which is available to authorized users.

## Background

Subarachnoid hemorrhage (SAH) is a severe subtype of stroke with high mortality and morbidity; 25% of SAH patients died within 2 days, and cognitive and functional deficits present in about 20% of SAH survivors [1, 2]. In recent years, the importance of early brain injury has been concerned by researchers. Initial clinical severity after SAH has been shown to be the most important predictor for clinical outcomes in patients [3]. In addition, alleviating early brain injury has been reported to exert neuroprotective effects in SAH model [4–6].

Neuroinflammation is a well-recognized consequence of SAH and considered as an important contributor for early brain injury, cerebral vasospasm, and delayed brain injury after SAH [7, 8]. In addition, neuroinflammation is closely associated with functional outcome in SAH patients [9, 10]. Toll-like receptors (TLRs) belong to a large family of pattern recognition receptors that play a key role in inflammatory responses [11]. Of all the TLR family members, TLR4 is widely expressed in the central nervous system, including microglia, neurons, astrocytes, endothelial cells [12]. After stimulation of TLR4 with ligands, the MyD88-dependent pathway activates NF-κB, which produces pro-inflammatory cytokines mediators such as tumor necrosis factor (TNF-α) [13]. Thus, therapies target TLR4 signaling pathway, and subsequent neuroinflammation may offer potential treatment to protect against neuroinflammation after SAH.

Fluoxetine is one of the serotonin selective reuptake inhibitors. Since the least toxicity and side effects, fluoxetine has been widely prescribed depression and anxiety disorders [14]. Neuroprotective effects of fluoxetine have been demonstrated in different neurological diseases [15–18]. Fluoxetine has been shown to exert a capacity to regulate neuroinflammation [19, 20]. Our recent study also demonstrated that fluoxetine inhibited NLRP3 inflammasome activation and subsequent necrotic cell death in early brain injury after SAH [18]. Importantly, recent studies also demonstrated that fluoxetine inhibits TLR4 activation and subsequent NF-κB signaling pathway in vivo and in vitro [21–24]. In the present study, we investigated the effects of fluoxetine in neuroinflammation and the potential TLR4/MyD88/NF-κB signaling pathway in early brain injury after SAH.

## Methods

### Study design

#### Experiment 1

Male SD rats were randomly divided into four groups: sham, sham+fluoxetine, SAH+vehicle, and SAH+fluoxetine group. Fluoxetine was purchased from Selleck and dissolved in sterile 0.9% NaCl. Fluoxetine (10 mg/kg) or vehicle was injected intravenously at 6 h after SAH induction as previously described [18]. SAH grade and neurological scores were measured in all groups. Brain edema (*n* = 6/group), Evans blue extravasation (*n* = 6/group), immunofluorescence/TUNEL staining (*n* = 5/group), quantitative real-time polymerase chain reaction (qRT-PCR) (*n* = 5/group), and western blot (*n* = 6/group) were performed.

#### Experiment 2

Male SD rats were randomly divided into four groups: SAH+vehicle, SAH+TAK-242, and SAH+fluoxetine, SAH+fluoxetine+TAK-242 group. TAK-242 (1.5 mg/kg), an exogenous TLR4 antagonist, was injected intraperitoneally 1 h after SAH as previously described [25]. Neurological scores, Evans blue extravasation (*n* = 6/group), qRT-PCR (*n* = 5/group), and western blot (*n* = 6/group) were performed.

### SAH model

Male Sprague-Dawley (SD) rats were purchased from SLAC Laboratory Animal Company (Shanghai, China) and housed in a controlled humidity and temperature conditions. The endovascular perforation model was performed to induce rat SAH as previously described [26]. Briefly, we isolated the left carotid artery and its branches under anesthesia of pentobarbital (50 mg/kg). Then, we divided the external carotid artery (ECA) and advanced a 4-0 monofilament suture until resistance was felt. Subsequently, we punctured the vessel and induced SAH. The sham rats underwent the same procedure without puncturing.

### SAH grade and neurological scores

SAH grade and neurological scores were blindly assessed at 24 h after SAH as previously described [27]. Briefly, the basal cistern was divided into six segments. Each part was blindly obtained a grade from 0 to 3 judging by the amount of the blood clot in subarachnoid space. Then, the rats have received a total score ranging from 0 to 18. Neurological scores were blindly evaluated with a modification of the Garcia scoring system [28]. This scoring system has six parts as follow: spontaneous activity, spontaneous movements of all limbs, movement of forelimbs, climbing wall of the wire cage, reaction to touch on both side of trunk, and response to vibrissae touch. Then, the rats have received a total score ranging from 3 to 18. In this study, rats with SAH grade < 9 were excluded.

### Brain edema and blood-brain barrier disruption

Brain edema and blood-brain barrier (BBB) disruption were evaluated at 24 h after SAH as previously described [29]. Brain water content and Evans blue leakage were used to assessing brain edema and BBB disruption. Briefly, under deep anesthesia, rats were sacrificed. Then, brains were removed and divided into the left hemisphere, right hemisphere, cerebellum, and brainstem. The left hemispheres were weighed immediately to get the wet weight and dried at 105 °C for 3 days to obtain dry weight. Brain water content was calculated as [(wet weight-dry weight)/wet weight] × 100%. Evans blue dye (2%, 5 ml/kg) was administrated via the left femoral vein and circulated for 1 h. Under deep anesthesia, rats were sacrificed by cardiac perfusion. Then, we removed and separated the brain to get the left hemispheres immediately. Subsequently, we weighted the brain samples and homogenized with 3 ml of 50% trichloroacetic acid, then centrifuged at 15000 g for 30 min. The supernatant was mixed with an equal volume of trichloroacetic acid with ethanol. After overnight incubation (4 °C), the samples were centrifuged again (15,000*g*, 30 min) and measured by spectrofluorophotometer (excitation wavelength 620 nm and emission wavelength 680 nm).

### Quantitative real-time polymerase chain reaction

The left basal cortical specimen (about 50–100 mg) in the face of the blood clot was collected for PCR detection at 24 h after SAH (as shown in Additional file 1: Figure S1). The total mRNA was then extracted using TRIzolTM Plus RNA Purification Kit (#12183-555, Invitrogen, China). Then, we determined the quantity of the purified RNA using UV absorbance at 260 nm. Subsequently, 1 μg of purified RNA from each sample was reverse-transcribed to cDNA. Superscript™ III First-Stand Synthesis SuperMix for qRT-PCR (#11752-050) was used to synthesize cDNA. The specific sequence of primers used was described as follows: TNF-α: sense primer 5′-GGT CCC AAC AAG GAG GAG AAG TTC-3′, antisense primer 5′-CCG CTT GGT GGT TTG CTA CGA C-3′; IL-1β: sense primer 5′-CGT GGG ATG ATG ACG ACC TGC-3′, antisense primer 5′-GGA GAA TAC CAC TTG TTG GCT TAT-3′; IL-6: sense primer 5′-GAC AGC CAC TGC CTT CCC TAC TT-3′, antisense primer 5′-CAG AAT TGC CAT TGC ACA ACT CT-3′; CD86: sense primer 5′-CAT CTA AGC AAG GAT ACC CGA AAC-3′, and antisense primer 5′-GAG ATA GGC TGA TGG AGA CAC TGA A-3′. PCR amplification was performed with a program of 95 °C for 1 min, followed by 40 cycles of 95 °C for 15 s, and 63 °C for 25 s. The relative mRNA level of each target gene was calculated using the 2^-∇∇CT^ methods as previously described [30].

### Immunofluorescence staining

Immunofluorescence staining was performed at 24 h after SAH as previously described [29]. Briefly, rats were deeply euthanized and perfused with 4% paraformaldehyde in 0.1-mM phosphate-buffered saline (PBS, PH7.4). Brain samples were immersed in 30% sucrose until sinking to the bottom; 18 um-thick slices were cut with a cryostat. The primary antibodies were polyclonal goat anti-Iba-1(1:500, ab5076, Abcam), monoclonal mouse anti-NeuN (1:500, ab104224, Abcam), and polyclonal rabbit anti-MPO (1:300, ab65871, Abcam). Alexa Fluor 594 donkey anti-goat IgG (1:500, Invitrogen) and donkey anti-mouse (1:500, Invitrogen) were used as secondary antibody. Terminal deoxynucleotide transferase-deoxyuridine triphosphate (dUTP) nick end labeling (TUNEL) was performed following the manufacturer’s protocol (Roche, Switzerland). Finally, the slices were covered by DAPI and observed under a fluorescence microscope. All procedures were conducted by two investigators blind to the experimental condition.

To quantify Iba-1-positive, MPO-positive, TUNEL-positive cells, we selected at least three sections per rats with similar areas of ipsilateral cortex (Additional file 1: Figure S1) and three fields with a magnification of × 200 or × 400 per section. For quantification of Iba-1-positive and MPO-positive cells, the numbers from these fields were averaged and expressed as positive cells per square millimeter for each mouse. For quantification of apoptotic neurons, the percentage of TUNEL-positive neurons was calculated as follows: (number of TUNEL-positive neurons/total number of neurons) × 100%. Tissue sections were analyzed by an observer who was blinded to the experimental cohorts.

### Western blot

Western blot was performed at 24 h after SAH as previously described [26]. Under deep anesthesia, rats were sacrificed by cardiac perfusion with 0.1 M PBS, then brains were removed and the left basal cortical specimens in the face of the blood clot were obtained. The nuclear proteins were prepared by the NE-PER nuclear extraction reagents (Thermo, Rockford). Briefly, the ipsilateral cortex was homogenized and centrifuged for 10 min (1000*g*, 4 °C). Total protein was determined by BCA Protein Assay Kit (Beyotime, Shanghai, China). An equal amount of protein (60 μg) was suspended in loading buffer (denatured at 95 °C for 5 min) and loaded on an SDS-PAGE and transferred to nitrocellulose membranes. Then, we blocked the membranes with a nonfat dry milk buffer for 2 h, followed by incubation overnight with the following primary antibodies: polyclonal rabbit anti-NF-κB p65 (1:1000, ab16502, Abcam), monoclonal mouse anti-TLR4 (1:200, sc-293,072, Santa Cruz Biotechnology), polyclonal rabbit anti-MyD88 (1:1000, ab2064, Abcam), monoclonal rabbit anti-MMP9 (1:5000, ab137867, Abcam), polyclonal rabbit anti-ZO-1 (1:2000, ab96587, Abcam); polyclonal goat anti-claudin-5 (1:500, sc-17,668, Santa Cruz Biotechnology), monoclonal rabbit anti-occludin (1:50000, ab167161, Abcam). The membranes were then incubated with horseradish peroxidase-conjugated secondary antibody for 1 h at room temperature. Blot bands were detected by X-ray film and quantified using Image J software (NIH).

### Statistical analysis

Data were presented as mean ± SD. For the data satisfying normal distribution, comparisons between groups were performed by one-way ANOVA as appropriate. For the data satisfying non-normal distribution, comparisons between groups were performed by Kruskal-Wallis test or Mann-Whitney test. Differences were considered significant at *p* < 0.05. All statistical analyses were performed using GraphPad Prism and SPSS software.

## Results

### Physiological parameters, mortality, and SAH grade

**Fig. 1:**
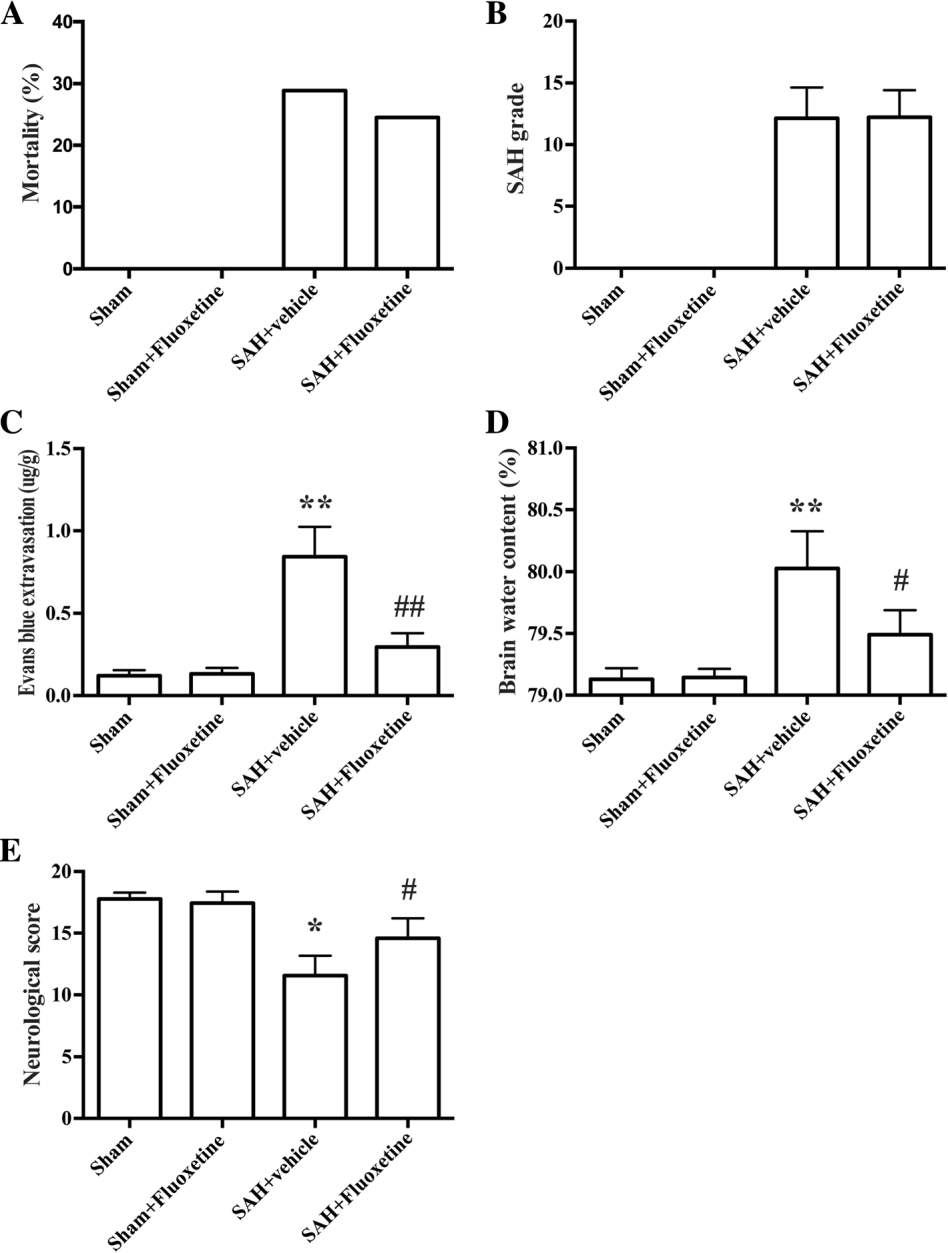
The effects of fluoxetine on mortality, SAH grade, neurological scores, Evans blue extravasation, and brain edema. **a** The quantification of mortality. **b** The quantification of SAH grade. *n* = 28/group. **c** The quantification of Evans blue extravasation. *p < 0.05 vs sham. ^**#**^*p* < 0.05 vs SAH+vehicle. *n* = 6/group. **d** The quantification of brain water content. **p < 0.01 vs sham. ^**#**^*p* < 0.05 vs SAH+vehicle. *n* = 6/group. **e** The quantification of neurological score. *p < 0.05 vs sham. ^#^*p* < 0.05 vs SAH+vehicle. *n* = 28/group

During the surgical procedures, no significant difference was observed in all physiological parameters including the mean arterial pressure, arterial PH, PO_2_, PCO_2_, and blood glucose levels (data not shown). None of the rats died in the sham and sham+fluoxetine group. In experiment 1, the mortality of SAH+vehicle and SAH+fluoxetine group were 28.2% (11/39, Fig. 1a) and 24.5% (9/37, Fig. 1a), respectively. In experiment 2, the mortality of SAH+vehicle, SAH+TAK-242, SAH+fluoxetine, and SAH+fluoxetine+TAK-242 group were 29.2% (7/24), 22.7% (5/22), 22.7% (5/22), and 19.0% (4/21), respectively. SAH grade was blindly evaluated in our study. However, there is no significant difference between SAH groups (*p* > 0.05, Fig. 1b).

### Fluoxetine attenuated BBB disruption, brain edema, and neurological deficits

SAH induction increased the amount of Evans blue extravasation, indicating the disruption of BBB (*p* < 0.01, Fig. 1c). Similarly, SAH also increased the ratio of brain water content, indicating brain edema (*p* < 0.01, Fig. 1d). In addition, neurological scores were lower in the SAH+vehicle group than in the sham rats, indicating neurological impairments (*p* < 0.01, Fig. 1e). The BBB disruption and brain edema were significantly attenuated by fluoxetine administration (*p* < 0.01, Fig. 1c and *p* < 0.05, Fig. 1d). Fluoxetine treatment also improved neurological function compared with the SAH+vehicle group (*p* < 0.05, Fig. 1e).

### Fluoxetine downregulated MMP-9 expression and decreased the degradation of tight junction proteins

**Fig. 2:**
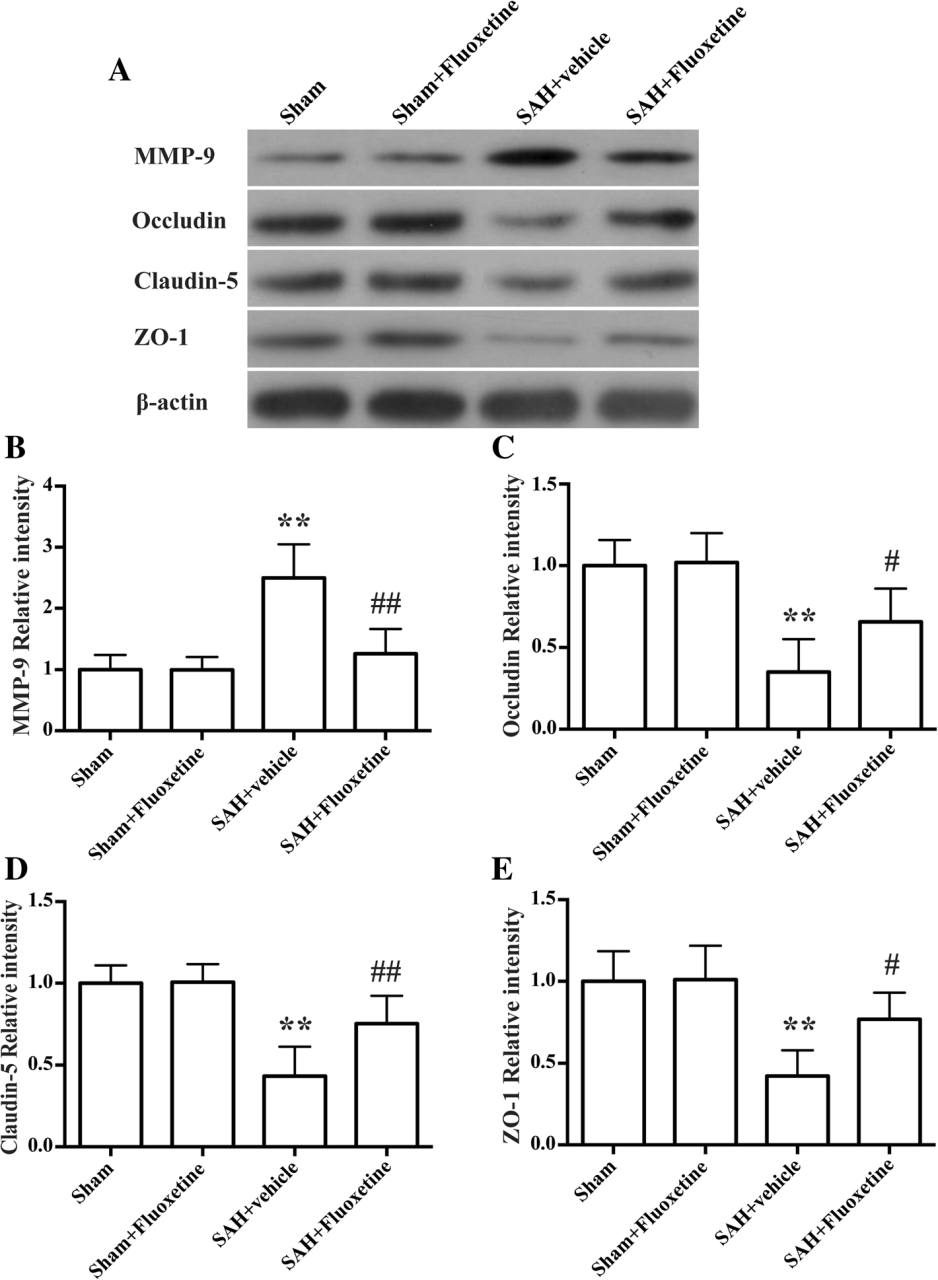
Fluoxetine downregulated MMP-9 expression and prevented degradation of the tight junction proteins in the ipsilateral cortex at 24 h after SAH. **a** Representative western blot bands of MMP-9, occludin, claudin-5, and ZO-1. **b** Densitometric quantification of MMP-9. **p < 0.01 vs sham. ^##^*p* < 0.01 vs SAH+vehicle. *n* = 6/group. **c** Densitometric quantification of occludin. **p < 0.01 vs sham. ^**#**^*p* < 0.05 vs SAH+vehicle. *n* = 6/group. **d** Densitometric quantification of claudin-5. **p < 0.01 vs sham. ^##^*p* < 0.01 vs SAH+vehicle. *n* = 6/group. **e** Densitometric quantification of ZO-1. **p < 0.01 vs sham. ^#^*p* < 0.05 vs SAH+vehicle. *n* = 6/group

Rats in SAH+vehicle group had high levels of MMP-9 than control rats (*p* < 0.01, Fig. 2a, b), and rats post-treated with fluoxetine had a lower MMP-9 expression (*p* < 0.01, Fig. 2a, b). The protein levels of tight junction proteins, including occludin, claudin-5, and ZO-1, were significantly decreased in the SAH+vehicle group when compared with the sham group (*p* < 0.01, Fig. 2 a, c–e), whereas fluoxetine treatment significantly upregulated their protein levels (*p* < 0.05, Fig. 2c, e, and *p* < 0.01, Fig. 2d).

### Fluoxetine decreased the expression of pro-inflammatory cytokines

**Fig. 3:**
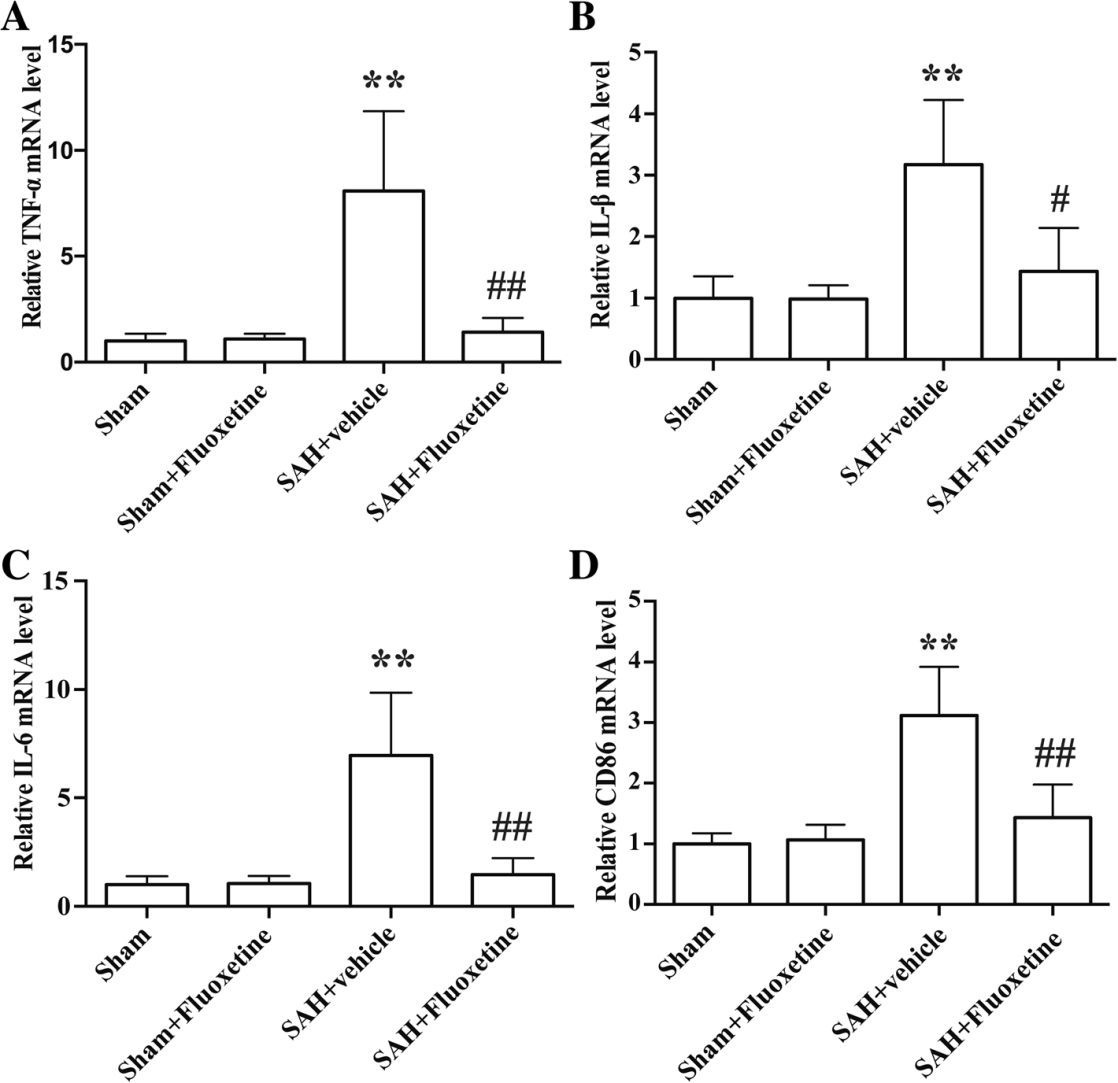
Fluoxetine decreased the mRNA levels of pro-inflammatory cytokines. **a** The quantification of TNF-α mRNA levels. **p < 0.01 vs sham. ^##^*p* < 0.01 vs SAH+vehicle. *n* = 5/group. **b** The quantification of IL-1β mRNA levels. **p < 0.01 vs sham. ^#^*p* < 0.05 vs SAH + vehicle. *n* = 5/group. **c** The quantification of IL-6 mRNA levels. **p < 0.01 vs sham. ^##^*p* < 0.01 vs SAH+vehicle. *n* = 5/group. **d** The quantification of CD86 mRNA levels. **p < 0.01 vs sham. ^##^*p* < 0.01 vs SAH+vehicle. *n* = 5/group

qRT-PCR results showed the mRNA levels of pro-inflammatory cytokines TNF-α, IL-1β, IL-6, and CD86 were significantly increased in the SAH+vehicle group compared with the sham group (*p* < 0.01, Fig. 3a–d), while fluoxetine administration significantly reduced the mRNA levels of TNF-α (*p* < 0.01, Fig. 3a), IL-1β (*p* < 0.05, Fig. 3b), IL-6 (*p* < 0.01, Fig. 3c), and CD86 (*p* < 0.01, Fig. 3d).

### Fluoxetine reduced Iba-1-positive microglia/macrophages, neutrophil infiltration, and neuronal apoptosis

**Fig. 4:**
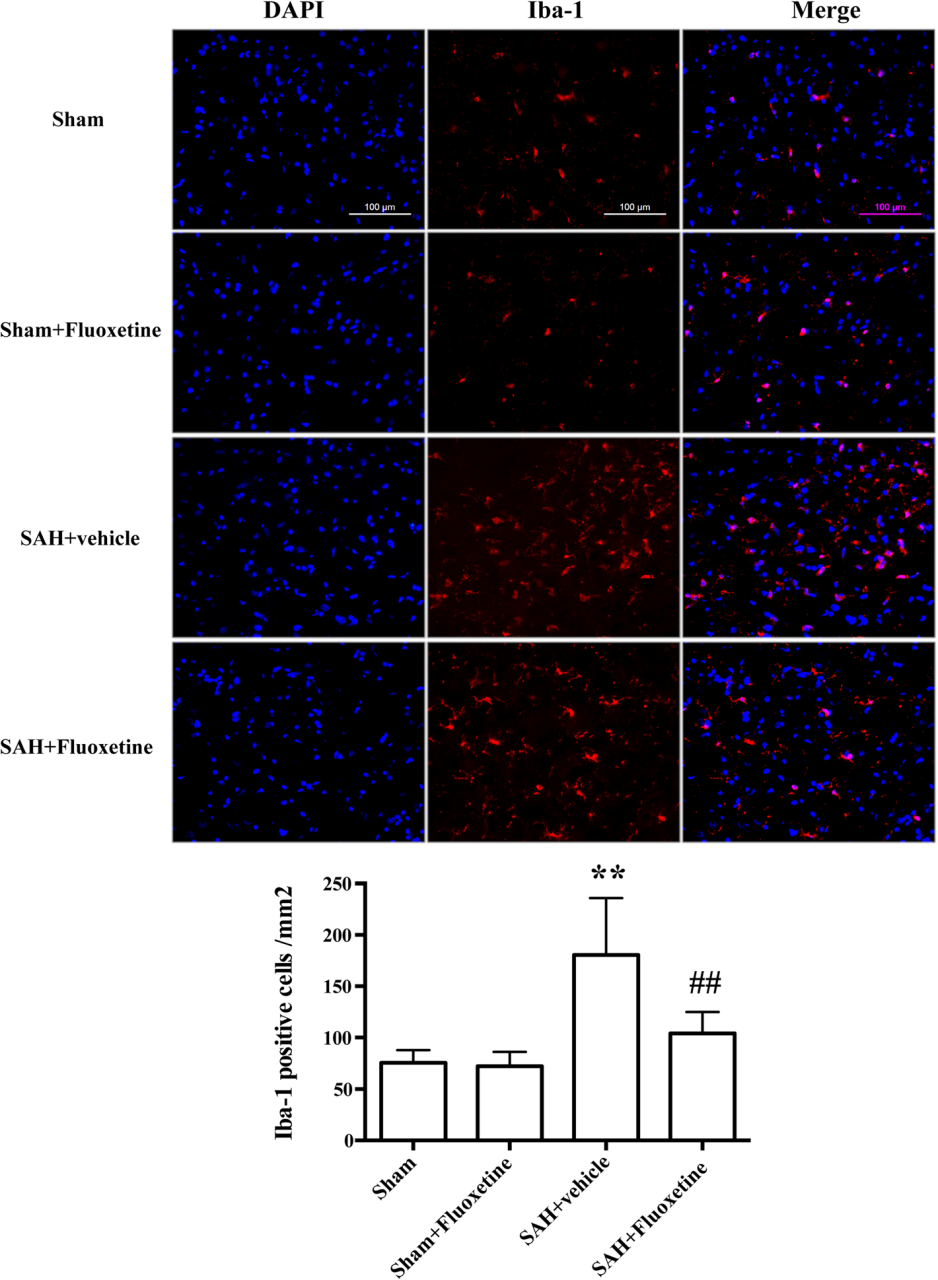
Fluoxetine decreased the number of Iba-1-positive microglia/macrophages in the ipsilateral cortex at 24 h after SAH. **a** Representative Iba-1 staining. **b** The quantification of Iba-1-positive cells. **p < 0.01 vs sham. ^##^*p* < 0.01 vs SAH+vehicle. Scale bar = 100 μm. *n* = 5/group

**Fig. 5:**
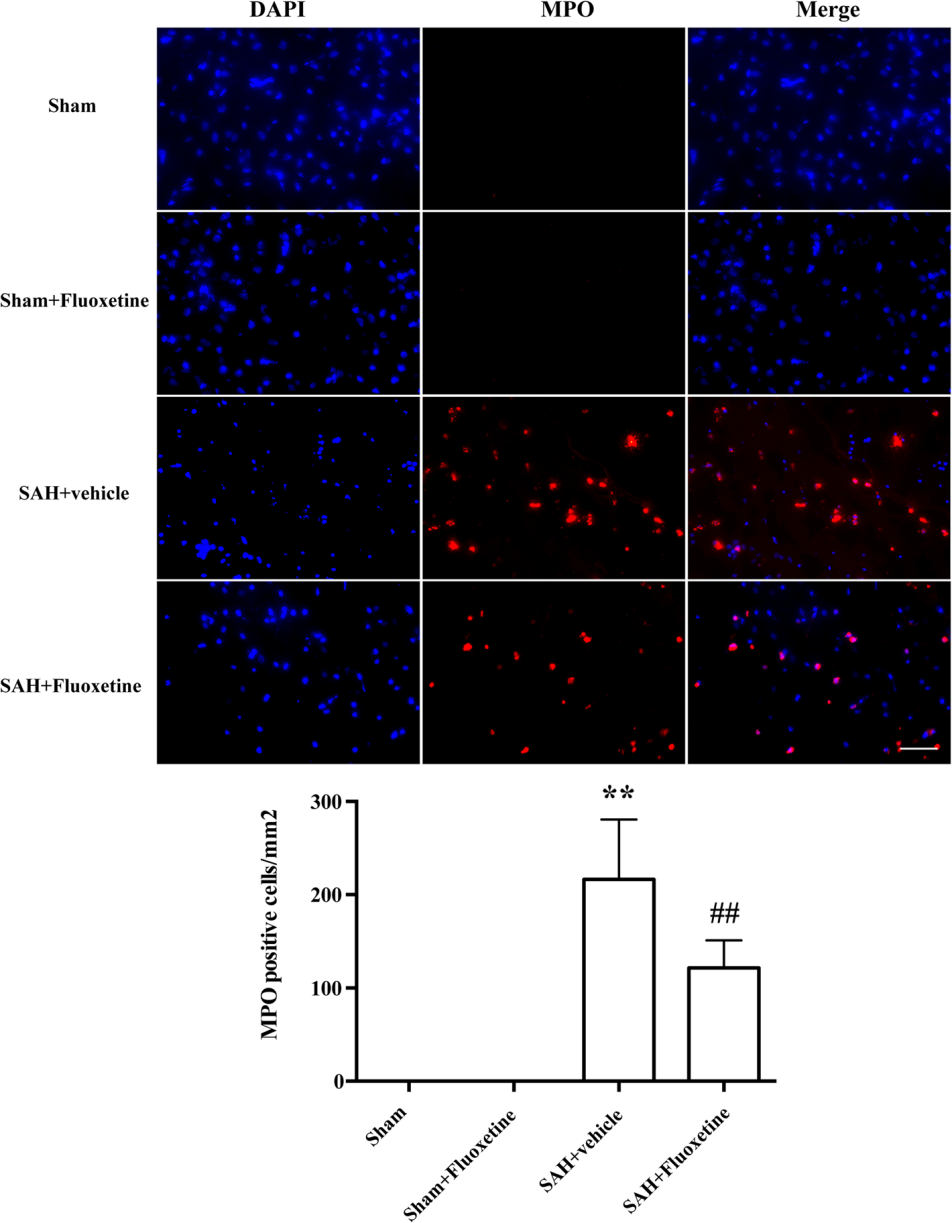
Fluoxetine inhibited neutrophil infiltration in the ipsilateral cortex at 24 h after SAH. **a** Representative MPO staining. **b** The quantification of MPO-positive cells. **p < 0.01 vs sham. ^##^*p* < 0.01 vs SAH+vehicle. Scale bar = 50 μm. *n* = 5/group

**Fig. 6:**
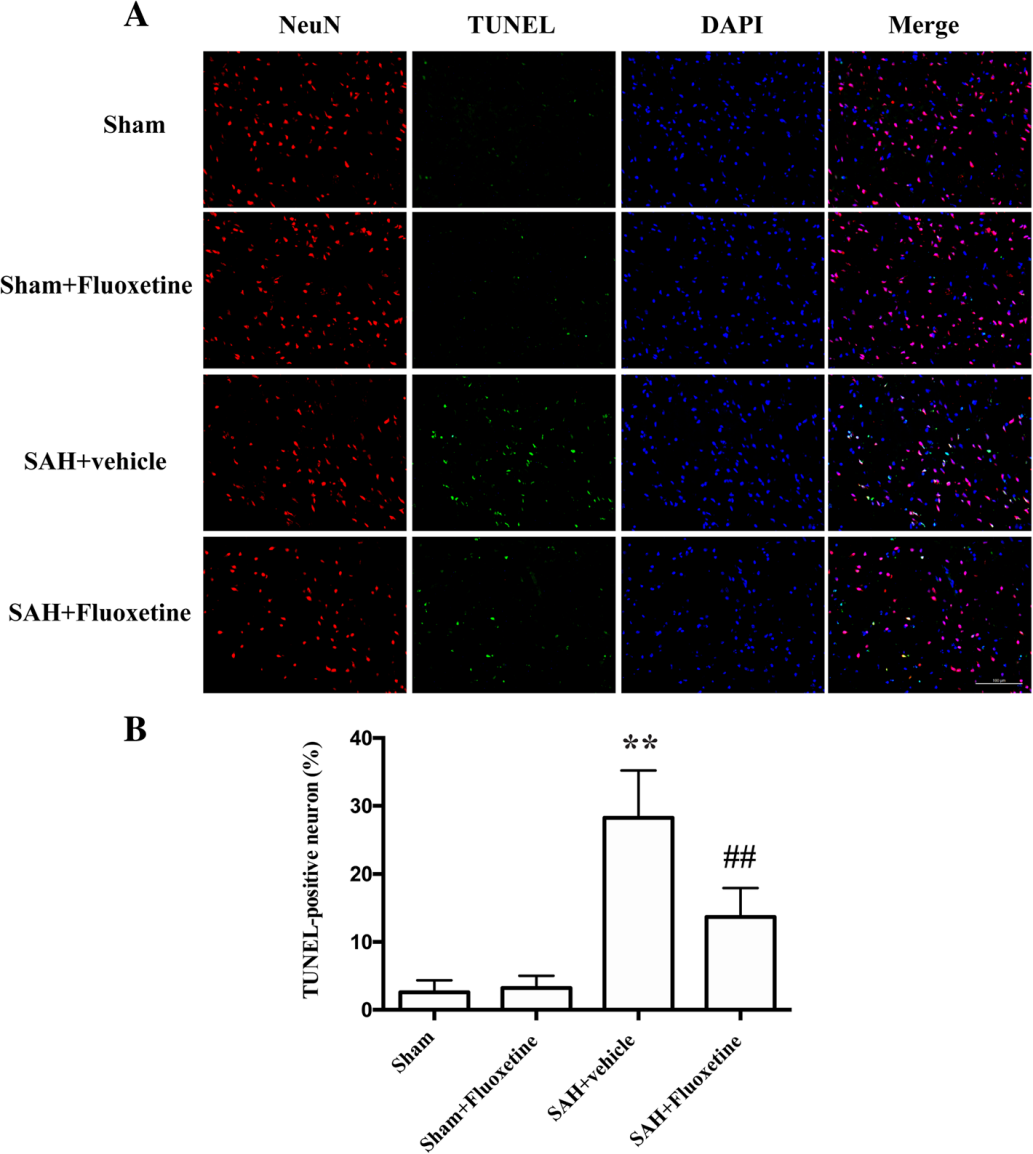
Fluoxetine attenuated neuronal apoptosis in the ipsilateral cortex at 24 h after SAH. **a** Representative TUNEL staining. **b** The percentage of TUNEL-positive neurons. ***p < 0.01 vs sham. ^##^*p* < 0.01 vs SAH+vehicle. Scale bar = 100 μm. *n* = 5/group

A significant increase of Iba-1positive cells was detected in SAH+vehicle group compared with the sham group (*p* < 0.01, Fig. 4a, b), whereas fluoxetine significantly decreased the number of Iba-1 positive cells (*p* < 0.01, Fig. 4a, b). MPO-positive cells markedly increased in the ipsilateral cortex after SAH when compared with the sham group (*p* < 0.01, Fig. 5a, b), while administration of fluoxetine significantly reduced the number of MPO-positive cells after SAH (*p* < 0.01, Fig. 5a, b). The percentage of TUNEL-positive neurons was significantly higher in the SAH+vehicle group than the control group (*p* < 0.01, Fig. 6a, b), whereas fluoxetine administration significantly decreased the percentage of TUNEL-positive neurons (*p* < 0.01, Fig. 6a, b).

### Fluoxetine attenuated TLR4 and MyD88 and reduced the nuclear translocation of NF-κB p65

**Fig. 7:**
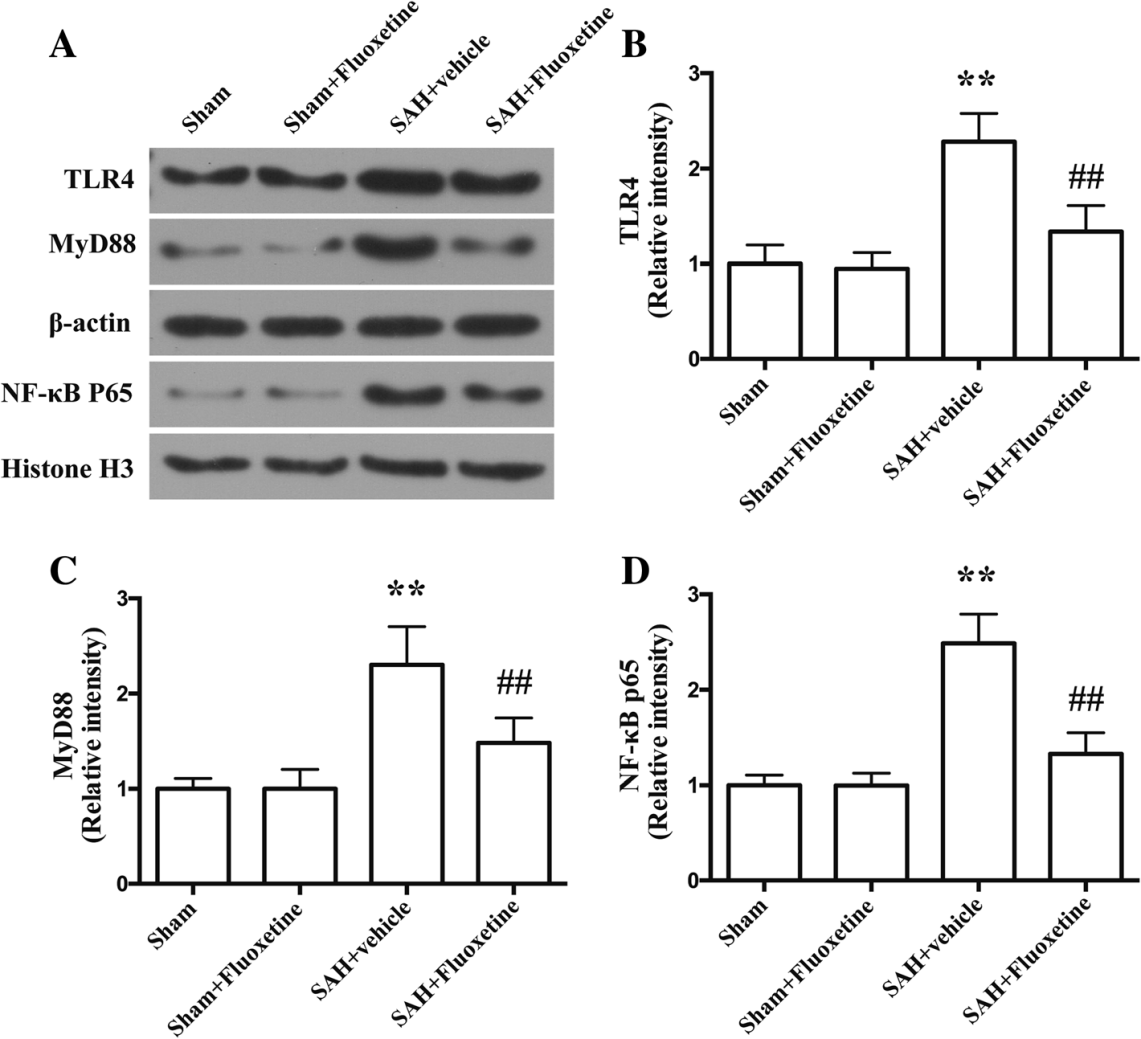
Fluoxetine downregulated the protein levels of TLR4 and MyD88, but increased the nuclear protein levels of NF-κB p65 in the ipsilateral cortex at 24 h after SAH. **a** Representative western blot bands of TLR4, MyD88, and nuclear NF-κB p65. **b** Densitometric quantification of TLR4. **p < 0.01 vs sham. ^##^*p* < 0.01 vs SAH+vehicle. *n* = 6/group. **c** Densitometric quantification of MyD88. **p < 0.01 vs sham. ^##^*p* < 0.01 vs SAH+vehicle. *n* = 6/group. **d** Densitometric quantification of nuclear NF-κB p65. **p < 0.01 vs sham. ^##^*p* < 0.01 vs SAH+vehicle. *n* = 6/group

The protein levels of TLR4 and MyD88 were significantly increased in the cortex in the SAH+vehicle group as compared with the sham group (*p* < 0.01, Fig. 7a–c). The protein levels of TLR4 and MyD88 in SAH+fluoxetine group were significantly lower than those of SAH+vehicle group (*p* < 0.01, Fig. 7a–c). We also examined the nuclear levels of NF-κB p65, an indicator of the activation of the NF-κB signaling pathway. The protein levels of NF-κB p65 were significantly increased in the SAH+vehicle group as compared with the sham group (*p* < 0.01, Fig. 7a, d). The protein levels of NF-κB p65 in SAH+fluoxetine group were significantly lower than those of SAH+vehicle group (*p* < 0.01, Fig. 7a, d).

### Combined administration of fluoxetine and TAK-242 did not enhance the effects of fluoxetine in the expression of TLR4, MyD88, nuclear NF-κB, and pro-inflammatory cytokines, BBB disruption, and neurological function

**Fig. 8:**
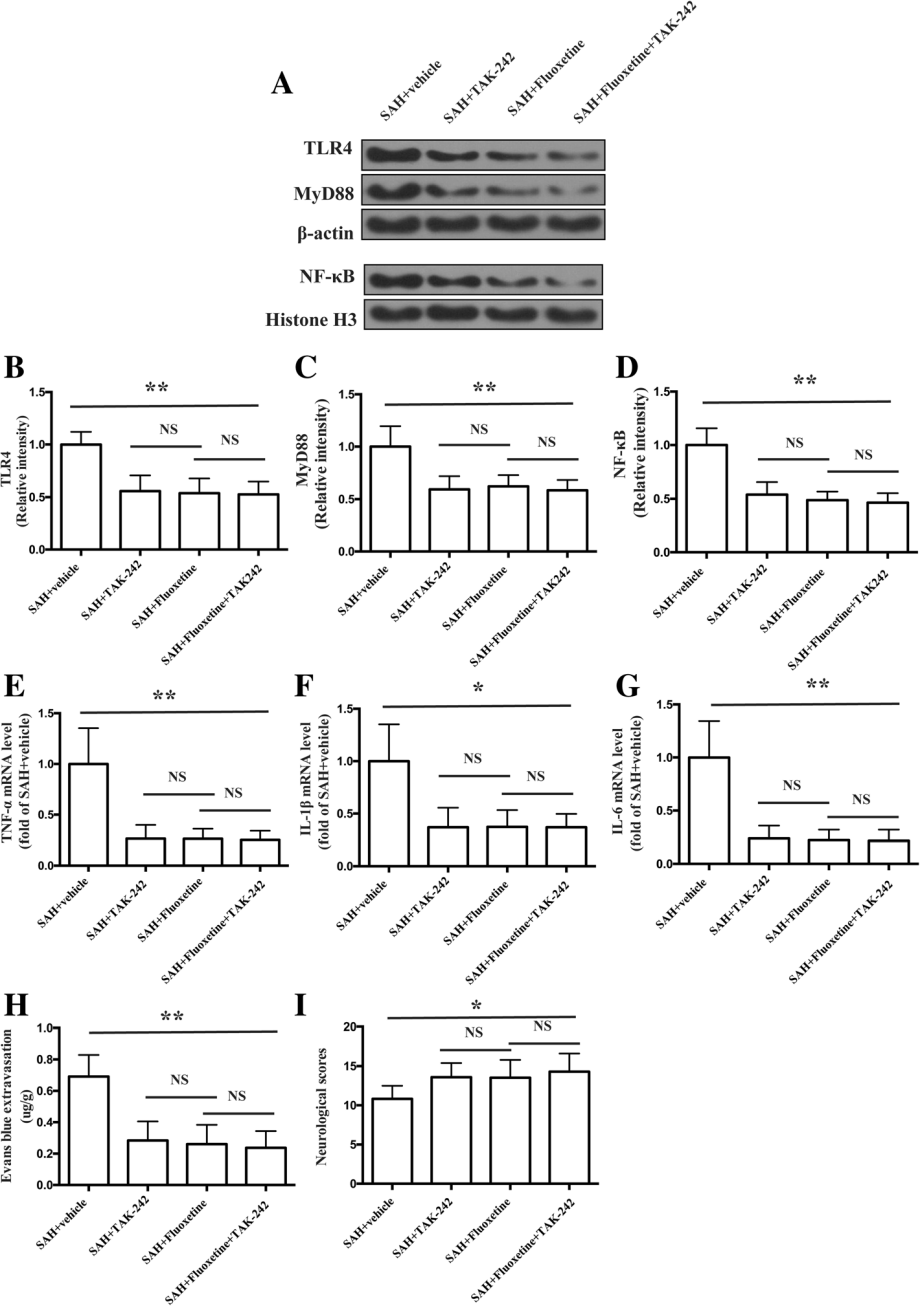
Combined administration of fluoxetine and TAK-242 did not enhance the effects of fluoxetine in the expression of TLR4, MyD88, nuclear NF-κB, and pro-inflammatory cytokines, BBB disruption, and neurological function. **a** Representative western blot bands of TLR4, MyD88, and nuclear NF-κB p65. **b** Densitometric quantification of TLR4. **p < 0.01 vs SAH+vehicle. *n* = 6/group. **c** Densitometric quantification of MyD88. **p < 0.01 vs SAH+vehicle. *n* = 6/group. **d** Densitometric quantification of nuclear NF-κB p65. **p < 0.01 vs SAH+vehicle. *n* = 6/group. **e** The quantification of TNF-α mRNA levels. **p < 0.01 vs SAH+vehicle. *n* = 5/group **f** The quantification of IL-1β mRNA levels. *p < 0.05 vs SAH+vehicle. *n* = 5/group. **g** The quantification of IL-6 mRNA levels. **p < 0.01 vs SAH+vehicle. *n* = 5/group. **h** The quantification of Evans blue extravasation. **p < 0.01vs SAH+vehicle. *n* = 6/group. **i** The quantification of neurological score. *p < 0.05 vs sham. ^#^*p* < 0.05 vs SAH + vehicle. *n* = 17/group

The protein levels of TLR4, MyD88, and nuclear NF-κB were significantly decreased in the cortex in the SAH+TAK242, SAH+fluoxetine, and SAH+fluoxetine+TAK-242 group as compared with the SAH+vehicle group (*p* < 0.01, Fig. 8a–d). There was no significant difference between SAH+fluoxetine and SAH+fluoxetine+TAK-242 in proteins level of TLR4, MyD88, and nuclear NF-κB (*p* > 0.05, Fig. 8a–d). The mRNA levels of pro-inflammatory cytokines TNF-α, IL-1β, and IL-6 were significantly decreased in the cortex in the SAH+TAK242, SAH+fluoxetine, and SAH+fluoxetine+TAK-242 group as compared with the SAH+vehicle group (*p* < 0.01, Fig. 8e, *p* < 0.05, Fig. 8f, and *p* < 0.01, Fig. 8g). No significant difference was observed in these mRNA levels between SAH+fluoxetine and SAH+fluoxetine+TAK-242 group (*p* > 0.05, Fig. 8e–g). Combined administration of fluoxetine and TAK-242 prevented the Evans blue extravasation (*p* < 0.01, Fig. 8h) and improved the neurological function (*p* < 0.05, Fig. 8i), which was similar with fluoxetine treatment (*p* > 0.05, Fig. 8h, i).

## Discussion

**Fig. 9:**
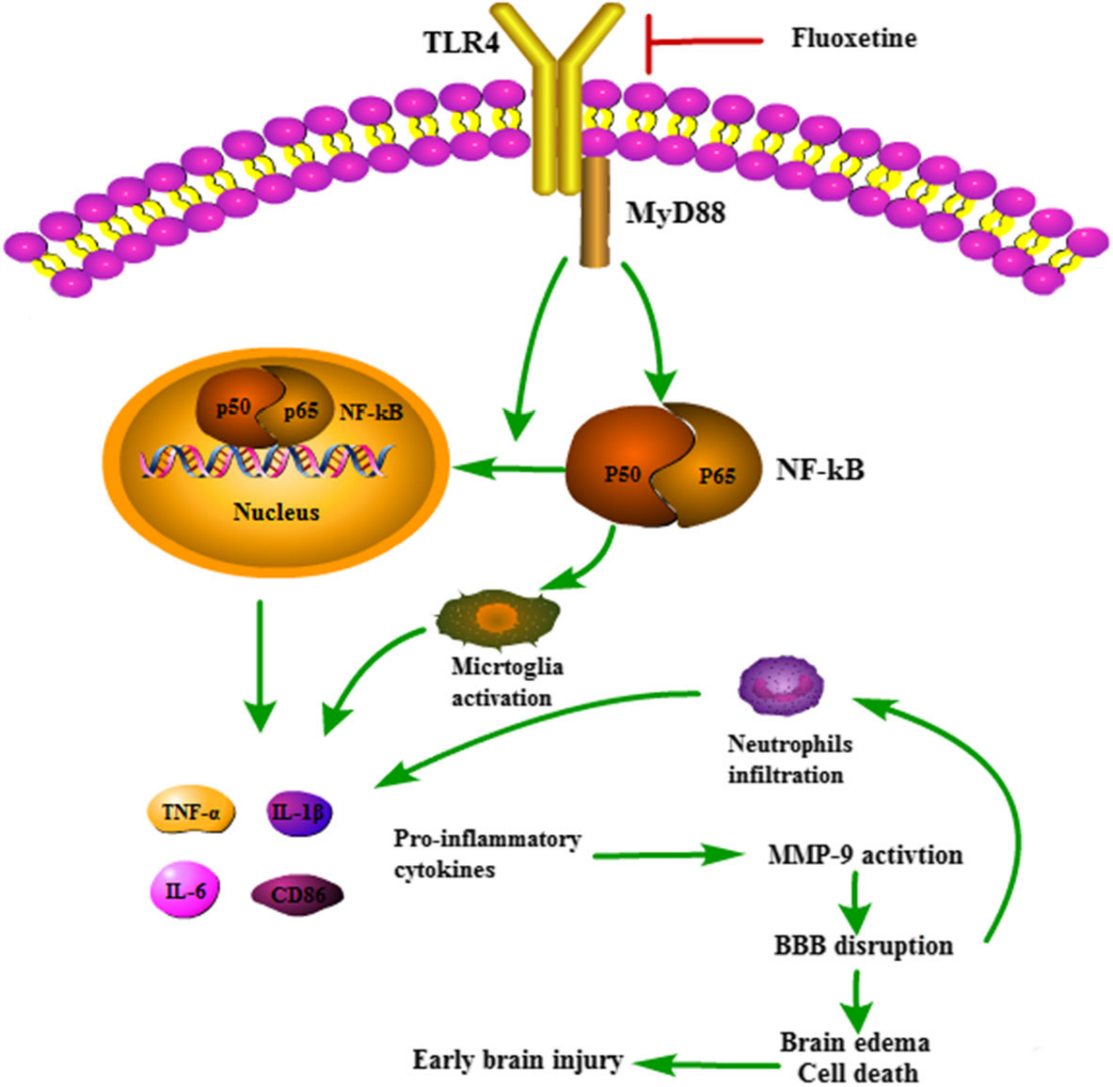
The potential molecular mechanisms of anti-inflammatory effects of fluoxetine through TLR4/MyD88/NF-κB signaling pathway

The current study presented several novel findings:(1) fluoxetine attenuated BBB disruption, brain edema, and improved neurological function after SAH. (2) Fluoxetine alleviated the number of Iba-1-positive microglia/macrophages, neutrophil infiltration, and cell death. (3) Fluoxetine reduced the levels of pro-inflammatory cytokines, and the underlying mechanisms, at least in part, involved the TLR4/MyD88/NF-κB signaling pathway (Fig. 9).

At present, no therapies are available to cure the neurological deficits in SAH patients [31]; however, an increasing number of studies show that inflammation contributes to early brain injury after SAH and inhibition of inflammation can ameliorate brain injury after SAH [10, 32–34]. One of the most important mediators in inflammation-induced brain injury after SAH is MMP-9. Notably, both clinical and basic studies have reported an elevation of MMP-9 in brain tissue, serum, and cerebrospinal fluid after SAH [35–37]. The MMP-9 elevation is responsible for the degradation of tight junction proteins, which are critical in the maintenance of BBB integrity. In the present study, we observed fluoxetine treatment alleviated MMP-9 expression and degradation of tight junction proteins (ZO-1, occludin, and claudin-5), attenuated BBB disruption and brain edema, and improved neurological function after SAH. The effects of fluoxetine on MMP-9 expression and subsequent BBB disruption were consistent with previous reports about fluoxetine treatment in experimental transient global ischemia and spinal cord injury [38, 39]. BBB disruption facilitates infiltration of peripheral inflammatory cells, including macrophage and neutrophils, which aggravate neuroinflammation by releasing a multitude of inflammatory factors [40]. In addition, BBB disruption also results in neuronal cell death [38]. In the current study, we found that fluoxetine inhibited the neutrophils infiltration and reduced neural cell death in early brain injury after injury.

Inflammatory cytokines are important regulators in MMP-9 activation and subsequent BBB disruption. A previous study demonstrated that the selective inhibitor of IL-1β blocked JNK-mediated MMP-9 activation and improved neurological function in SAH rats [41]. IL-6 induced MMP-9 expression through the JAK-mediated pathway in macrophages [42]. TNF-α was also reported as an upstream regulator for MMP-9 [43]. In our study, we found that fluoxetine downregulated these inflammatory cytokines, which is in accordance with previous studies [44, 45]. TLRs belong to a large family of pattern recognition receptors that play a key role in innate immunity and inflammatory responses. Of the TLR family members, TLR4 was of vital importance in this family. TLR4 is activated by many endogenous ligands such as heme, fibrinogen, heat shock proteins, all of which are produced after SAH [13, 46]. In fact, patients with aneurysmal SAH was reported to have higher TLR4 levels on peripheral blood mononuclear cells, which were associated with more massive SAH, occurrence of cerebral vasospasm, delayed cerebral infarction, and worse functional recovery [47]. TLR4 interacts with two distinct adaptor proteins, MyD88 and Toll-receptor-associated activator of interferon (TRIF), and activates two parallel signaling pathways to initiate activation of transcription factors that regulate expression of proinflammatory cytokines genes [12]. In addition, the TRIF-dependent pathway induces late phase activation of NF-κB, while the faster TLR4 route through MyD88 is the early activation of NF-κB. Many previous studies have demonstrated the anti-inflammatory effects of fluoxetine in other system disorders in vivo and in vitro [20–24, 48]. In these studies, TLR4 and downstream NF-κB were hotspots. However, limited studies focused on the effects of fluoxetine in the TLR4 signaling pathway after SAH. Therefore, we examined the TLR4-mediated MyD88 pathway in the current study. Our results showed that the protein levels of TLR4 and MyD88 were significantly increased in early brain injury after SAH and fluoxetine downregulated their expression. In addition, we found that fluoxetine also reduced the nuclear translocation of NF-κB p65, an indicator of NF-κB activation. What is more important is that we also used TAK-242, a small-molecule inhibitor of the TLR4 signaling pathway. We found that the combined administration of fluoxetine and TAK-242 reduced the expression of TLR4, MyD88, and nuclear NF-κB, decreased the mRNA levels of pro-inflammatory cytokines, prevented BBB disruption, and improved neurological function after SAH. These beneficial effects of combined administration were similar to alone fluoxetine treatment. Taken together, our study indicated that TLR4/MyD88/NF-κB signaling pathway was involved in anti-inflammatory effects of fluoxetine in early brain injury after SAH.

There are some limitations in our study. First, the present study aimed at investigating the effects and potential mechanism of fluoxetine in neuroinflammation in early brain injury after SAH, the long-term study of fluoxetine after SAH is still needed in the future. Second, previous studies showed anti-apoptotic effects of fluoxetine, which did not deeply evaluate in the current study.

## Conclusions

The current study has demonstrated that fluoxetine attenuated neuroinflammation and improved neurological function after SAH. The potential mechanisms involved, at least in part, TLR4/MyD88/NF-κB signaling pathway.

## Additional file

Additional file 1: **Figure S1.** Representative pictures of brains in SAH group showing the sample region. (TIF 372 kb)

## Abbreviations

BBB: Blood-brain barrier
ECA: External carotid artery
IL-1β: Interleukin-1β
IL-6: Interleukin-6
MMP-9: Matrix metallopeptidase 9
MPO: Myeloperoxidase
MyD88: Myeloid differentiation primary response 88
NF-κB: Nuclear factor kappa B
qRT-PCR: Quantitative real-time polymerase chain reaction
SAH: Subarachnoid hemorrhage
SD: Sprague-Dawley
TLRs: Toll-like receptors
TNF-α: Tumor necrosis factor-α
TUNEL: Terminal deoxynucleotidyl transferase dUTP nick end labeling
