# Supplementary figures and images for: Fluoxetine attenuates neuroinflammation in early brain injury after subarachnoid hemorrhage: a possible role for the regulation of TLR4/MyD88/NF-κB signaling pathway
Source: J Neuroinflammation. 2018 Dec 20;15:347. doi: 10.1186/s12974-018-1388-x (PMC6302437; doi:10.1186/s12974-018-1388-x)

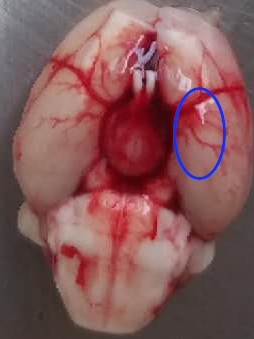

Supplement: Supplementary file 1 — Figure S1. Representative pictures of brains in SAH group showing the sample region. (TIF 372 kb) [file 12974_2018_1388_MOESM1_ESM.tif]
